# Involvement of the glymphatic/meningeal lymphatic system in Alzheimer’s disease: insights into proteostasis and future directions

**DOI:** 10.1007/s00018-024-05225-z

**Published:** 2024-04-23

**Authors:** Kaoru Yamada, Takeshi Iwatsubo

**Affiliations:** 1 Department of Neuropathology, Graduate School of Medicine, The University of Tokyo, Tokyo, Japan; 2 National Institute of Neuroscience, National Center of Neurology and Psychiatry, Kodaira, Tokyo Japan

**Keywords:** Brain waster clearance, CNS lymphatics, ISF, CSF, Neurodegenerative diseases

## Abstract

**Background:**

Alzheimer’s disease (AD) is pathologically characterized by the abnormal accumulation of Aβ and tau proteins. There has long been a keen interest among researchers in understanding how Aβ and tau are ultimately cleared in the brain. The discovery of this glymphatic system introduced a novel perspective on protein clearance and it gained recognition as one of the major brain clearance pathways for clearing these pathogenic proteins in AD. This finding has sparked interest in exploring the potential contribution of the glymphatic/meningeal lymphatic system in AD. Furthermore, there is a growing emphasis and discussion regarding the possibility that activating the glymphatic/meningeal lymphatic system could serve as a novel therapeutic strategy against AD.

**Objectives:**

Given this current research trend, the primary focus of this comprehensive review is to highlight the role of the glymphatic/meningeal lymphatic system in the pathogenesis of AD. The discussion will encompass future research directions and prospects for treatment in relation to the glymphatic/meningeal lymphatic system.

## Introduction

AD is a progressive neurodegenerative disorder that primarily affects memory, cognitive functions, and behavior. It stands as the most prevalent cause of dementia among older adults. Estimates indicated that the number could increase significantly due to factors such as an aging population and improved diagnostics, and there is a pressing need to develop effective treatment approaches. The pathology characterizing AD is marked by two types of abnormal proteins: Aβ and tau. The proper concentration of these proteins is maintained by proteostasis, and dysregulation particularly in the clearance system are believed to disrupt this equilibrium, leading to the accumulation of these proteins as fibrils and ultimately increasing the risk of developing AD. In addition to the local clearance system, the involvement of global waste clearance through the glymphatic/meningeal lymphatic system has been strongly suggested in the removal of Aβ and tau. Moreover, histological and imaging analyses have suggested a dysfunction in the glymphatic system in AD, which could be one of the contributing factors to the accumulation of Aβ and Tau. In this review, we describe the involvement of glymphatic/meningeal lymphatic system in clearance of Aβ and tau, factors involved in their regulation and disease-associated changes occurred during the pathogenesis of AD. Finally, we address unresolved questions and explore future research challenges in this field.

### Glymphatic/meningeal lymphatic systems

**Fig. 1:**
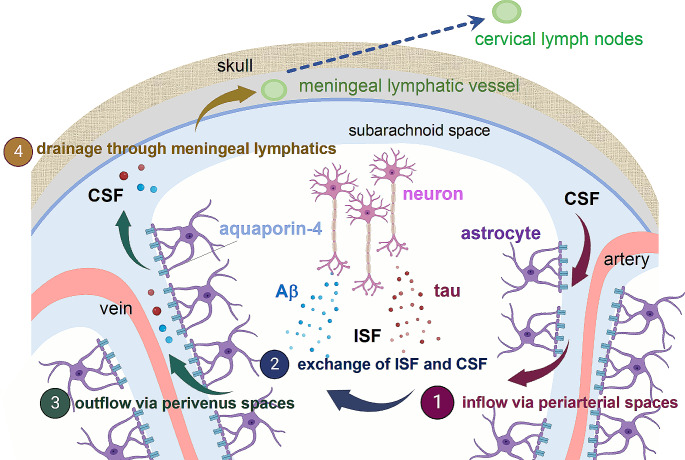
Glymphatic/Meningeal lymphatic clearance in relation to AD. In the glymphatic system, it is hypothesized that CSF inflows into brain parenchyma from the subarachnoid space through the periarterial spaces, where it mixes with ISF. Throughout this process, Aβ and Tau released from neurons into the ISF are thought to be collected and subsequently exit the bran through outflow via perivenous spaces. The molecules that reach the CSF are then drained through lymphatic vessels present in the dura mater and transported to cervical lymph nodes. Created partly with BioRender.com

Central nervous system (CNS) is supported by two forms extracellular fluid, interstitial fluid (ISF) and cerebrospinal fluid (CSF). ISF fills the intercellular spaces in the brain, serving as a primary compartment for receiving secretions from brain cells while CSF is a fluid present in the brain ventricles and the subarachnoid space. The concept of the glymphatic system involves the directional flow of these extracellular fluid within the brain. Utilizing two-photon microscopy, Nedergaard group revealed that CSF travels from the subarachnoid space through the perivascular space of arteries, enters the ISF, flushes out metabolic waste products, and then returns to the subarachnoid space through the perivascular space of veins [1] (Fig. 1). The biological function of glymphatic system is to remove metabolic waste products and toxins from the brain, helping to maintain brain health and function. It becomes particularly active during sleep, allowing efficient clearance of waste that accumulates throughout the day. It has been suggested that it is due to an increase in the interstitial space in the brain during sleep, allowing more efficient clearance of waste products [2].

The glymphatic system is not a distinct anatomical structure like blood vessels or organs but rather a functional system that involves the movement of extracellular fluids through the brain’s interstitial space. One of the driving forces of the convective flows of this glymphatic system is thought to be the arterial pulsations associated with the cardiac cycle [3, 4]. Additionally, it works together with perivascular astrocytes expressing the water channel aquaporin-4 (AQP4), aiding in the circulation of CSF throughout the brain tissue. Due to the significant reduction in glymphatic clearance caused by the deficiency of AQP4, AQP4 knockout mice are commonly employed as a model for glymphatic dysfunction. The term ‘’glymphatic’’ was coined due to the essential role of astrocytes and the functional resemblance to peripheral lymphatics, combining “glia” and “lymph”.

While the glymphatic system drains ISF to CSF, the pathway that drains substrates from CSF to the periphery was unclear. Historically, the CNS was widely believed to lack of a lymphatic system, which raised questions about how the brain managed protein clearance and immune responses. However, in 2015, groundbreaking studies conducted by two independent research groups unveiled the existence of lymphatic vessels within the dura mater, the outer most layer of the meninges [5, 6]. Using imaging techniques combined with molecular markers, these studies provided compelling evidence for the presence of functional lymphatic vessels in the dura, challenging the long-standing assumption that the CNS lacked a direct connection to the lymphatic system. The primary biological function of the meningeal lymphatic system is to serve as a conduit for the drainage of CSF and macromolecules from the CNS to the peripheral lymph nodes. This function contributes to maintaining brain health by preventing the accumulation of harmful substances and ensuring a balanced environment for neural function. In addition to drainage, meningeal lymphatic vessels are also essential for brain immune surveillance [7], although this aspect is not the primary focus of this review.

### The pathogenesis of AD and the roles of local clearance

AD is pathologically characterized by extracellular aggregation of Aβ plaque and intracellular accumulation of fibrillar tau protein [8]. Aβ accumulation represents the earliest pathological event in AD and also one of the primary causes of AD [9, 10]. This is strongly indicated by genetic mutations associated with familial AD, which are linked to the Aβ precursor APP and the Aβ-producing enzymes Presenilin-1 or 2 [11]. Aβ accumulation plays a pivotal role as the primary driver of tau aggregation [12], which is closely correlated with clinical symptoms and directly linked to neurodegeneration [13]. The aggregation of these proteins occurs in a concentration-dependent manner, meaning an imbalance between their clearance and production can trigger aggregation, thereby increasing the risk of developing AD. Due to Aβ being a secreted protein and tau being a cytoplasmic protein, their levels are maintained mostly in extracellular and intracellular compartments, respectively.

Aβ is generated through proteolytic cleavage from its precursor transmembrane protein, APP. After its production and secretion into the extracellular space, Aβ undergoes various clearance processes. Aβ is cleared through proteolytic degradation by enzymes [14, 15] or internalization into cells via phagocytosis or endocytosis via receptors such as LDL Receptor-Related Protein-1 (LRP-1). Microglia primarily undertake phagocytosis [16], but endocytosis has also been demonstrated in cells such as astrocytes and neurons [17, 18]. It has also demonstrated that soluble Aβ is actively transported into the bloodstream through transporters present in the blood-brain barrier (BBB) [19].

Inhibiting these pathways are shown to lead abnormal accumulation of Aβ [14, 15, 17, 18, 20], underscoring the role of these clearance pathways in regulating extracellular Aβ levels.

Unlike in dominant inhered AD with genetic mutations in APP or Presenilin-1 or 2, sporadic AD has not been associated with Aβ overproduction. In fact, the pulse-chase kinetic study using the stable isotope labeling kinetics (SILK) technique have demonstrated that in sporadic AD patients, despite the production rate of Aβ remaining unchanged, the clearance rate is significantly reduced [21].

The impact of AD risk factors on Aβ clearance serves as additional evidence of the link between clearance and AD development. Apolipoprotein E, a component of lipoprotein particles, with the ε4 isoform being the strongest genetic risk factor for AD, has been shown to reduce Aβ clearance possibly through competition to common cell surface receptors such as LRP-1 [22, 23]. This suggests that the reduction of Aβ clearance in the presence of apoEε4 is one contributing factor to the increased risk of AD onset.

In contrast to Aβ, tau is a cytoplasmic protein and is metabolized through intracellular degradation pathways. The ubiquitin-proteasome system (UPS) and the autophagy-lysosome system are recognized as two major clearance mechanisms for degrading tau [24]. In general, UPS is responsible for degrading soluble, short-lived proteins, while autophagy-lysosome system can degrade long-lived, aggregated protein [25]. Although tau forms insoluble, fibrillar aggregates, considerable levels of turnover of the fibrils have been documented in cells and in vivo [24, 26]. Deficits in UPS and autophagy-lysosome system have been both implicated in deposition of tau protein [27, 28], although the exact contribution of each pathway to tau proteostasis can depend on the cellular context and the aggregation status of tau protein.

Research on proteostasis in AD has primarily focused on local clearance mechanisms as mentioned above. However, with the discovery of the glymphatic/meningeal lymphatic system, there has been increasing attention to the involvement of global waste clearance in the removal of Aβ and tau.

### Glymphatic clearance of pathogenic proteins in AD

Following the demonstration by Iliff and colleagues that Aβ is cleared by the glymphatic system [1], its involvement in AD pathogenesis gained significant scientific attention. Subsequent studies demonstrated exacerbation of Aβ accumulation and associated neurofunctions in AQP4-deficient APP transgenic mice, suggesting that the glymphatic system is one of the major clearance pathways for Aβ [29, 30].

The perivascular localization of AQP4 around blood vessels is crucial for the proper glymphatic function, and it has been shown that a decrease in this localization leads to reduced glymphatic flow. AQP4-x is a translational readthrough variant with C-terminal extension that shows exclusive localization at perivascular astrocytes likely due to its stronger interaction with α-syntrophin [31]. The deletion of AQP4-x or α-syntrophin not only reduces perivascular AQP4 but also elevates Aβ levels in APP transgenic mice without altering total AQP4 expression levels [32, 33]. Those data collectively suggest that glymphatic clearance of Aβ assisted by perivascular AQP4 plays a significant role in Aβ metabolism.

The glymphatic system is a mechanism for the clearance of substances through the flow of extracellular fluid, thus it was long thought to not be directly involved in the removal of intracellular proteins. Tau, despite being a microtubule-binding protein, possesses the unique characteristic of physiological secretion into ISF [34, 35]. It has been demonstrated that both soluble and aggregated forms of tau protein are released into the extracellular space, involving pathways such as exosome and lysosomal exocytosis [36–39]. It has been shown that when aggregated tau is released into the extracellular space, it can be taken up by neighboring cells and propagate its aggregated state. Studies have demonstrated that the anti-tau antibodies can inhibit propagation and alleviate tau pathology, further indicating the critical role of extracellular tau in the propagation process [40]. Building upon these observations, Harrison et al., and we have demonstrated that tau is also subject to glymphatic clearance [41, 42]. We also found that in tau transgenic mice lacking AQP4, the accumulation of tau and neurodegeneration were exacerbated. This finding highlights that the glymphatic clearance can ultimately influence the levels of intracellular pathogenic proteins as well although the exact underlying mechanisms remain to be elucidated.

Aβ and tau could be degraded through different pathways depending on their aggregation states. However, previous research has primarily focused on the glymphatic clearance of monomeric Aβ and tau proteins, and the dynamics of polymeric/aggregated forms of these proteins within the brain remains to be explored.

### Disease- or age-associated changes in glymphatic system

Studies have demonstrated that Aβ and tau are both actively cleared by the glymphatic system and its dysfunction results in abnormal accumulation of these proteins. So, does the glymphatic impairment occur in AD brains? In this section, we present several papers that suggest the abnormalities in glymphatic system in AD.

First, there are reports suggesting that the accumulation of Aβ or tau itself may lead to disruptions in the glymphatic system. The localization of AQP4 at perivascular astrocyte end foot processes is crucial for the proper function of glymphatic system, and there have been reports that it is altered in AD and mouse models of Aβ deposition [43, 44]. Similarly, in mouse models that accumulate tau in an age-dependent manner, changes in AQP4 vascular localization and impaired glymphatic system function have been proposed [41, 45]. Cerebral amyloid angiopathy (CAA) is characterized by the accumulation of Aβ in the walls of cerebral blood vessels [46]. This accumulation weakens the blood vessel walls, making them prone to damage and potentially leading to hemorrhages or microbleeds in the brain. CAA is commonly associated with AD, as Aβ plaques also accumulate in the brain parenchyma. It has been shown that CAA can disrupt the normal functioning of the glymphatic system [47]. This can lead to an increase in Aβ levels in the brain, potentially contributing to the further progression of CAA and AD.

Glymphatic system was initially described in rodent brain but subsequent studies using magnetic resonance imaging (MRI) have suggested its presence in human CNS [48, 49]. In addition to the significant reduction of water transport observed in AD brains compared to control [50], certain non-coding SNPs found in AQP4 have been reported to influence the rate of cognitive decline following AD diagnosis [51].

Furthermore, there are reports suggesting that risk factors for AD can lead to dysfunction of glymphatic system abnormalities. Sleep disturbances and aging are robust environmental risk factors for AD. Interestingly both are also closely associated with dysfunction of glymphatic system. As described earlier, during sleep, the glymphatic system becomes highly active while it is suppressed during wakefulness. This observation aligns with data showing that levels of Aβ and tau in ISF increase during wakefulness and decrease during sleep, and that sleep deprivation significantly exacerbates Aβ and tau pathology [52, 53].

During the natural aging process, there are alterations in the structure and function of the glymphatic system. Studies have demonstrated that in middle-aged rodents, arterial pulsatility and the expression of polarized AQP4 are reduced [54]. These changes could potentially be an underlying factor contributing to the altered efficiency of glymphatic clearance.

### The involvement of meningeal lymphatic system on clearance of pathogenic proteins in AD

Studies have been conducted using mouse models exhibiting Aβ pathology or tau pathology to investigate how the decline of the lymphatic system impacts AD pathogenesis. A study has shown that ablation of meningeal lymphatic vessels using photodynamic drugs aggravates Aβ pathology in APP transgenic mice [55]. It has been also shown that tau clearance from brain is significantly delayed in transgenic mice that lack functional CNS lymphatic vessels [56]. These studies have collectively suggested that drainage of CSF through dural lymphatics is important as a final exit route for the clearance of Aβ and tau produced in ISF.

An alternative approach to block drainage via the lymphatic system and study its consequences is through surgical ligation of lymphatics that reach cervical lymph nodes. Consistent with the findings mentioned earlier, it has been shown that deep cervical lymph node ligation also exacerbates Aβ pathology, neuroinflammation and cognitive behaviors [57].

Much like the glymphatic system, the presence of meningeal lymphatics in humans has also been demonstrated through studies employing MRI [58, 59]. The presence of Aβ in human lymph nodes also support the hypothesis that glymphatic/lymphatic system may contribute to Aβ clearance in human brains [60].

Cribriform plate and nasal mucosa also harbor functional lymphatics that drain into superficial cervical lymph nodes and considered as an exit route for immune cells and CSF [61]. There is a study reported that CSF tracer were exclusively effluxed along olfactory nerves through the cribriform plate to reach the nasal mucosa rather than dural lymphatics [62]. The contributions of the nasal or the meningeal dural lymphatics to CSF macromolecule drainage in the context of different CNS insults and diseases remains a topic of debate.

### Disease- or age-associated changes in lymphatic system

There are reports indicating that aging not only impairs the function of the glymphatic system but also that of the meningeal lymphatic system [63]. Furthermore, there are papers suggesting that this decline occurs not only in rodents but also in humans [64]. While further investigation is needed to understand the factors contributing to age-dependent lymphatic dysfunction a recent study conducting single-cell sequencing of FACS-enriched lymphatic endothelial cells from the dura of aged mice revealed chronic elevation of IFN-g is one responsible factor for reduced CSF drainage in aging brains [65].

The function of meningeal lymphatics declines not only with aging but also in various pathological conditions. For example, traumatic brain injury (TBI), a risk factor for AD, is known to exacerbate neuroinflammation by inducing a decline in meningeal lymphatic function [66].

## Future directions

Since the study by Iliff and colleagues, hundreds of papers on the glymphatic system have been published to date. However, certain aspects, such as the directional flow in the glymphatic system, have been called into question. One factor contributing to the controversy is the technical difficulties in measuring the fluid flow in the brain. The glymphatic system can be influenced by various physiological factors, including sleep [2], circadian rhythm [67], and anesthetic agents [68], and the fixation method of the brain is known to influence the structure of perivascular spaces critical for the glymphatic system [4]. Furthermore, the majority of the studies highly depend on AQP4-deficient mice as genetic models to study glymphatic system, but it’s important to consider that AQP4 may have other physiological functions beyond the glymphatic system [69].

There are also technical limitations in measuring the function of meningeal lymphatics. Various methods have been reported for inhibiting or enhancing meningeal lymphatics in rodent brains [55–57]. However, there are technical challenges associated with specifically manipulating meningeal lymphatics.

It is also crucial to conduct in-depth investigations into how clearance by glymphatic/meningeal lymphatic systems are regulated by specific molecules, cells, and the brain microenvironment. In addition to the pulsation of arteries and the perivascular localization of AQP4, various elements beyond those initially suggested at the discovery of the glymphatic system have now been reported. For instance, parenchymal border macrophages are shown to influence vascular pulsation presumably by participating in the metabolism of the extracellular matrix, thereby regulating CSF flow [70]. In line with this discovery, it has been demonstrated that the chemical ablation of parenchymal border macrophages leads to the increased accumulation of Aβ and tau, suggesting its crucial role in the progression of AD pathology [45, 70]. Additionally, aside from perivascular spaces, brain interstitial space is also important anatomical region for glymphatic fluid flows. Considering this perspective, the size of the interstitial space and the interactions between the extracellular matrix and substrates should influence its molecular diffusion and efficient flow. Glymphatic dysfunction occurs in aging and AD brains, but the exact mechanisms remain unclear. It is possible that changes in glymphatic function are not solely due to the direct impact of protein accumulation but also due to factors such as astrogliosis/microgliosis and disease-associated alterations in the extracellular environment.

Studies have revealed that C-C chemokine receptor type 7 (CCR7) is expressed in T cells and is reduced in aging mice. Intriguingly, its deletion resulted in a decrease in perivascular AQP4 levels and glymphatic flows, accompanied by enhanced Aβ accumulation and microglial activation. This suggests a potential interplay between the glymphatic system and immune cells [71]. In addition, there is also a potential interaction between the meningeal lymphatics and brain resident phagocytes, particularly microglia in the clearance of Aβ [72]. Further research is needed to gain a better understanding of how glymphatic, lymphatic systems and immune cells interact each other and how the regulations of one system may influence the other.

It has become increasing evident that key molecules in AD, Aβ and tau are both drained by the glymphatic system and subsequently cleared through meningeal lymphatics. In addition, the pathogenesis and risk factors of AD are closely intertwined with the impairment of glymphatic/lymphatic function.

While the majority of current therapeutic interventions for AD primarily target Aβ and tau directly, one critical research challenge is to determine whether enhancement of brain endogenous clearance pathways could be also a therapeutic strategy.

To date, several regulatory factors that enhance glymphatic/lymphatic clearance have been identified. For example, there is data suggesting that voluntary exercise enhances glymphatic flow although the underlying mechanisms for this phenomenon remain unclear [73]. Vascular endothelial growth factor-C (VEGF-C) elicits lymphangiogenesis by binding to its receptor vascular endothelial growth factor receptor-3 (VEGFR-3), which is highly expressed on lymphatic endothelial cells. It has been shown that VEGF-C followed by mouse analog of Aducanumab enhances meningeal lymphatic function and improve Aβ clearance [72]. While these experimental results are promising, it would be necessary to verify whether these regulations identified in rodent brains are also applicable to humans for future treatment strategies.

Additionally, it’s worth considering that dural lymphatics not only play a crucial role in the removal of macromolecules but also immune surveillance in CNS. Multiple types of immune cells present in the meninges have been shown to play a role in the certain disease condition. Of note, higher number of T cells has been reported in CSF of individuals with AD [74] and the infiltration of cytotoxic T cells is implicated in the pathogenesis of AD, particularly neurodegeneration caused by tau protein [75]. Apart from the function of macromolecule clearance, how changes in meningeal lymphatics impact these immune cells in CNS is also crucial.

Finally, the glymphatic/meningeal lymphatic system may also hold potential significance in considering the value of AD diagnostic biomarkers. Once produced in the cells, Aβ and tau are cleared from ISF by multiple pathways and subsequently reach CSF and bloodstream. Recent advances in highly sensitive mass spectrometry and immunoassays have made it possible to reliably measure Aβ and tau levels in the blood, providing promising, less-invasive indicators of AD brain pathology [76, 77]. However, the levels of Aβ and tau in ISF, CSF and blood may not always correlate [34, 78, 79], suggesting the need to better understand how these proteins levels are regulated within their respective compartments.　 Questions such as the primary pathway for draining these proteins into the bloodstream and how dysfunction of glymphatic/meningeal lymphatic functions affects their levels are also important considerations for future research.

Although AD heterogeneity needs to be considered when interpreting the influences of glymphatic/meningeal lymphatic dysfunction on the progression of AD, addressing these remaining questions should be a crucial step towards therapies targeting the glymphatic/meningeal lymphatic system and provide important insights for diagnosis of AD as well.

## Data Availability

Not applicable.
